# Unsupervised electric signal separation for linking behavior and electrocommunication in *Gnathonemus petersii*

**DOI:** 10.1038/s41598-025-18291-5

**Published:** 2025-09-29

**Authors:** Ivana Chrtkova, Vlastimil Koudelka, Veronika Langova, Jan Hubeny, Petra Horka, Karel Vales, Roman Cmejla, Jiri Horacek

**Affiliations:** 1 National Institute of Mental Health, Klecany, Czech Republic; 2 Faculty of Electrical Engineering, Czech Technical University in Prague, Prague, Czech Republic; 3 Third Faculty of Medicine, Charles University, Prague, Czech Republic; 4 Institute for Environmental Studies, Faculty of Science, Charles University, Prague, Czech Republic

**Keywords:** *Gnathonemus Petersii*, Electrocommunication, Unsupervised learning, Sonification, Schizophrenia, Ketamine, Electrophysiology, Software

## Abstract

The transfer of information between individuals is fundamental to living systems and requires comprehensive research in various species. Weakly electric fish, *Gnathonemus petersii*, provides a unique model organism for such investigations due to its advanced electrocommunication via electric organ discharges (EODs). As separating EODs from multiple individuals remains challenging, we developed an unsupervised approach for EOD separation in two free-swimming individuals. Using continuous wavelet transform, t-distributed Stochastic Neighbor Embedding, and hierarchical clustering, we achieved accurate discrimination of EODs without the necessity of any training data. This approach overcomes the supervised algorithms based on previously published methods in accuracy and computational efficiency, simplifies experimental procedures, and supports animal well-being by reducing the number of required measurements. We applied our separation approach in a dyadic fish model, where ketamine was used to induce schizophrenia-like behavior in one fish. We confirmed the ketamine-induced alteration of the intrinsic relationship between locomotion and EOD signaling. Moreover, while ketamine-induced changes in locomotion were socially transferred, correlated changes in EOD signaling were not observed between dyad members, which may be interpreted as a communication deficit. Additionally, we introduced two techniques for EOD sonification, facilitating exploratory analysis of EOD sequences. These advancements lay the groundwork for future studies of EOD-based communication, highlighting the potential of *Gnathonemus petersii* in neuroethological, psychopharmacological, and translational research.

## Introduction

Communication is the foundation of information flow, involving exchanging, transmitting, and interpreting signals within a social context. Animal models are essential for studying these processes and understanding the mechanisms underlying information transfer^1–3^. The weakly electric fish *Gnathonemus petersii* (*G. petersii*), capable of emitting and perceiving electric signals, preserves an ideal model for exploring communication principles in complex systems. The fish generates electric organ discharges (EODs) for electrolocation and electrocommunication, enabling navigation and interaction with conspecifics within its environment^4,5^. EODs are produced by an electric organ, located in the fish’s caudal peduncle^6^, which consists of electrocytes whose synchronized excitation generates EODs and establishes a three-dimensional electric field surrounding the fish^7,8^. The waveform characteristics carry information about an individual’s identity^9,10^, while the precise timing of discharges provides insights into the behavioral context and responses to external stimuli^9,11,12^. The analysis of inter-pulse intervals (IPIs) – the time intervals between consecutive EODs – and exploration of generated patterns offer valuable opportunities to elucidate how neural circuits encode and process sensory information, thereby contributing to deciphering the neural code^9^.

Recent studies suggest that *G. petersii* may represent a promising novel animal model for the research of psychiatric disorders, especially schizophrenia^13,14^. Schizophrenia is characterized by severe communication impairments, including disorganized thinking, poverty of speech, and disturbed social cognition. Previous studies have already shown that some aspects of social behavior in the context of schizophrenia can be examined in other model species, laboratory rodents, and *Danio rerio* fish^15–17^. However, *D. rerio* expresses only limited social behavior, and the signaling component in rodents is too complex (vocalizations, scent marking, and tactile communication) to be applicable in modelling language analog^18^. The idea that *G. petersii* can enrich the study of schizophrenia is based on their complex communication, which includes behavioral patterns accompanied by EOD as an electric signaling mechanism. It stems from the study of Kunze and Wezstein^19^, who demonstrated that EOD signaling can be pharmacologically modulated by apomorphine, an antagonist of dopaminergic receptors. These observations highlight the significance of *G. petersii* in translational research, addressing the limitations of standard animal models^18,20^.

EOD of *G. petersii* is characterized by a biphasic pulse pattern^21,22^ with a brief duration of approximately 200–400 µs^23,24^. The short pulse length presents a considerable challenge in developing classifiers capable of accurately separating EODs from multiple individuals recorded simultaneously. The frequency of EOD generation depends on the behavioral context and may exceed 100 Hz^24,25^. In social experiments, recordings often capture thousands of EODs from multiple individuals, making manual separation impractical and underscoring the need for automated algorithms to enhance the efficiency of signal processing.

With the advancement of machine learning methods, various sophisticated algorithms that solve this task with high accuracy have been developed^26–28^. However, these methods typically rely on supervised learning and require pre-recorded EODs from a single fish to create a training dataset for assigning EODs to corresponding individuals from recordings of dyads. A simple automated method (that does not require manual assignment) is based on the cross-correlation of EODs from dyadic recordings with templates obtained during the recordings of a single fish in the aquarium^29^. However, this approach is insufficient when individuals exhibit similar EOD durations or discharge almost at the same time. More advanced algorithms include the classification with support vector machine (SVM) utilizing extracted features from EODs^26,27^ or combining the waveform characteristics and positional information from video tracking data^28^. Although effective, these methods often require substantial computational resources or complex experimental setups. Additionally, the fish must undergo several measurements to obtain training data for classifiers, which may be stressful for individuals involved in the experiment. The accuracy of classification may also be limited since the training data originates from different recording sessions, introducing additional variability that cannot be explained by the model.

To remedy these pitfalls, our study aimed to develop a novel approach based on unsupervised learning for separating EODs from two free-swimming *G. petersii* fish. Before constructing the classifier, the inter-individual variability in EOD waveforms was explored to determine the most effective feature representations for capturing this diversity and achieving the highest classification accuracy. These features were visualized in a lower dimension with t-distributed Stochastic Neighbor Embedding (t-SNE) and subsequently classified using hierarchical clustering. The performance of the developed approach was compared with the classification results from the two algorithms based on supervised learning to investigate its effectiveness. To explore potential sources of performance variance, the influence of inter-individual differences in body parameters (e.g., weight, length) on the classification accuracy was investigated, utilizing these readily available non-invasive measures. In addition, we introduced an effective tool to integrate behavioral patterns with electric activity through sonification, providing an intuitive overview of the relationship between these two modalities.

Finally, the direct application of our unsupervised approach was demonstrated in the schizophrenia model. Regarding a recent glutamatergic theory of schizophrenia^20^, which has replaced the dopaminergic theory, we used the standard pharmacological approach utilizing the NMDA receptors antagonist ketamine^13,14,20^. This experiment follows our previous study, confirming the impact of ketamine on the number of generated EODs and its association with locomotion^13^. Experiments involving dyads of *G. petersii* were conducted to examine the effect of ketamine on the relationship between locomotion and EOD signaling, as well as to investigate how the presence of a ketamine-treated fish influences this relationship in untreated conspecifics. We hypothesized that the ketamine-induced state would alter this relationship, while leaving the locomotor and electric activity of untreated conspecifics unaffected. Additionally, the relationship between the direct effect of ketamine on treated fish and the indirect effect on untreated conspecifics was examined to assess the treatment’s impact on social behavior observed in both EOD and locomotion modalities.

## Results

### Datasets

**Fig. 1:**
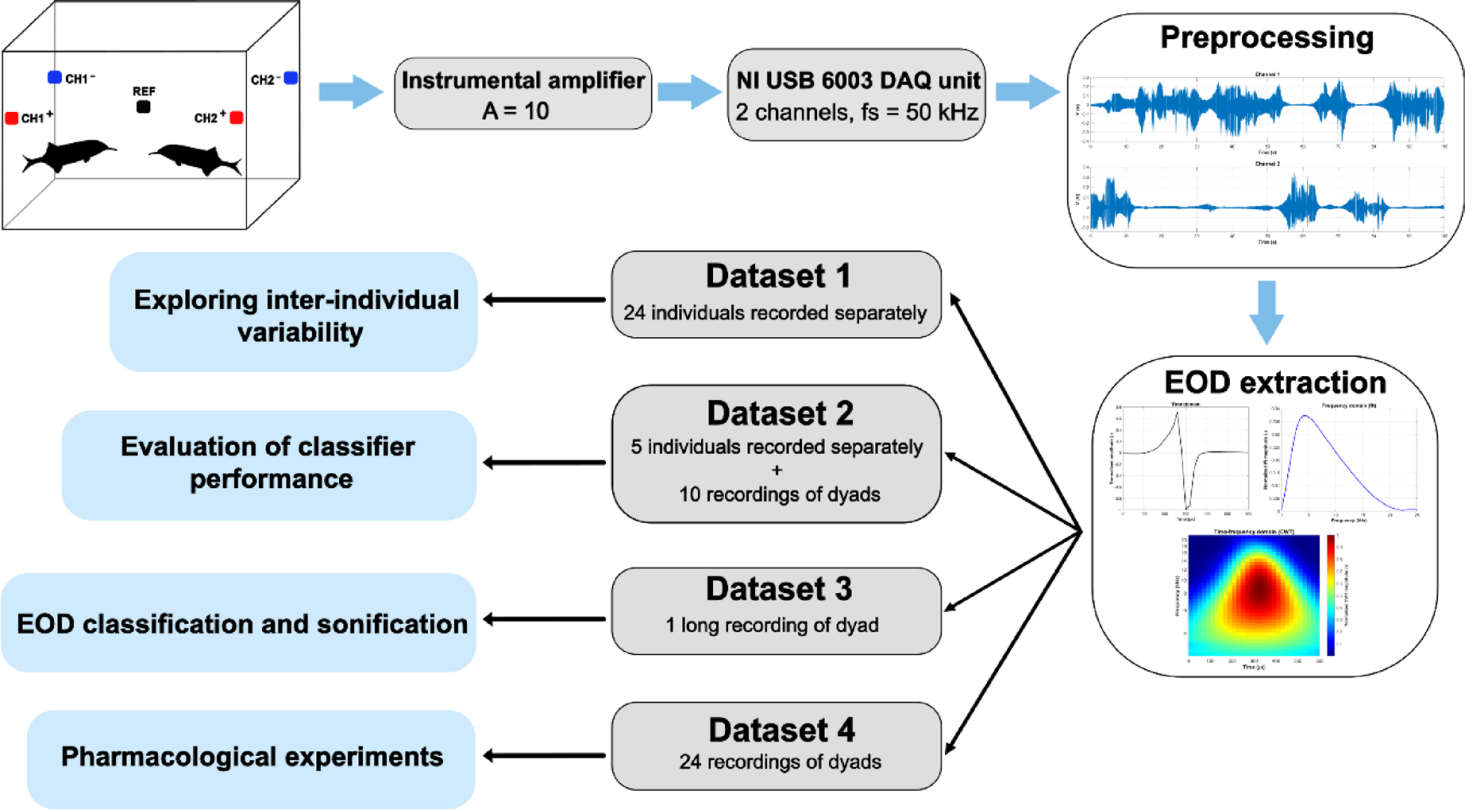
Diagram of recording setup and processing pipeline used in this study. Two channels were recorded with four active bipolar-connected electrodes and one electrode as a reference. Recorded signals were amplified and sampled with a sampling frequency of 50 kHz for each channel. Digitized signals were preprocessed and individual EODs were extracted utilizing 300 µs before and after the detected EOD and stored in the form of normalized waveforms in the time domain, normalized spectra of original waveforms using fast Fourier transform, and normalized scalograms obtained from continuous wavelet transform. Signals from four different datasets were used for exploration of inter-individual variability (Dataset 1), construction and validation of separation approaches: Correlation method, SVM, and t-SNE + hierarchical clustering (Dataset 2), classification and sonification of EODs (Dataset 3), and demonstration of EOD separation in social experiments with pharmacological manipulations (Dataset 4).

Four distinct datasets were acquired to develop, validate, and demonstrate the proposed EOD separation approach (Fig. 1). Dataset 1 comprises 24 separately recorded individuals to explore the inter-individual variability in EOD shapes. Dataset 2 includes recordings from five individuals recorded individually and in 10 dyadic combinations with manually labeled EODs. This dataset was used for training and validation of EOD separation approaches. Dataset 3 consists of a single long recording of a dyad, employed to illustrate the EOD sonification. Dataset 4 was used to demonstrate our EOD separation approach in schizophrenia research. It consists of 24 dyadic recordings involving pharmacologically treated individuals (Active group, *n* = 12) paired with one from untreated conspecifics (Control, *n* = 3). Recordings include baseline sessions where both members of the dyad were untreated (Baseline condition), followed by sessions in which individuals from the Active group were treated with ketamine, while individuals from the Control group remained untreated (Ketamine condition). This dataset enabled investigation of ketamine’s effects on the relationship between locomotion and EOD signaling in social experiments.

## Exploring inter-individual variability of EODs

**Fig. 2:**
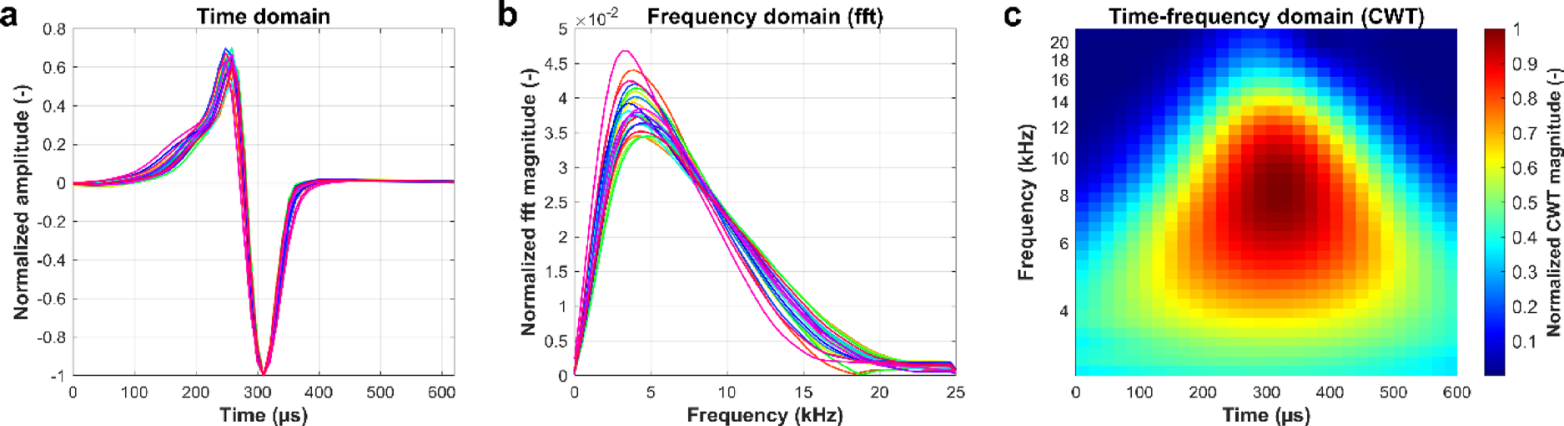
Illustration of three different representations of EODs for exploration of inter-individual variability. a, Averaged EODs for 24 individuals from Dataset 1 in the time domain (interpolated for visualization), (**b**), frequency domain - obtained by fast Fourier transform of averaged EODs, and (**c**), time-frequency domain - obtained by continuous wavelet transform of grand average from 24 individuals. In (**a**) and (**b**), each individual is represented by a different color label, with colors assigned in accordance with Fig. 3a-c.

The first step in our analysis was to explore inter-individual variability in the EOD waveforms across 24 individuals recorded separately (Dataset 1). EODs were represented in three different domains (Fig. 2) – time domain (original EOD), frequency domain (fast Fourier transform), and time-frequency domain (continuous wavelet transform) – and used as the input to the t-SNE algorithm to find the most accurate representation for capturing differences in EOD shapes among individuals.

**Fig. 3:**
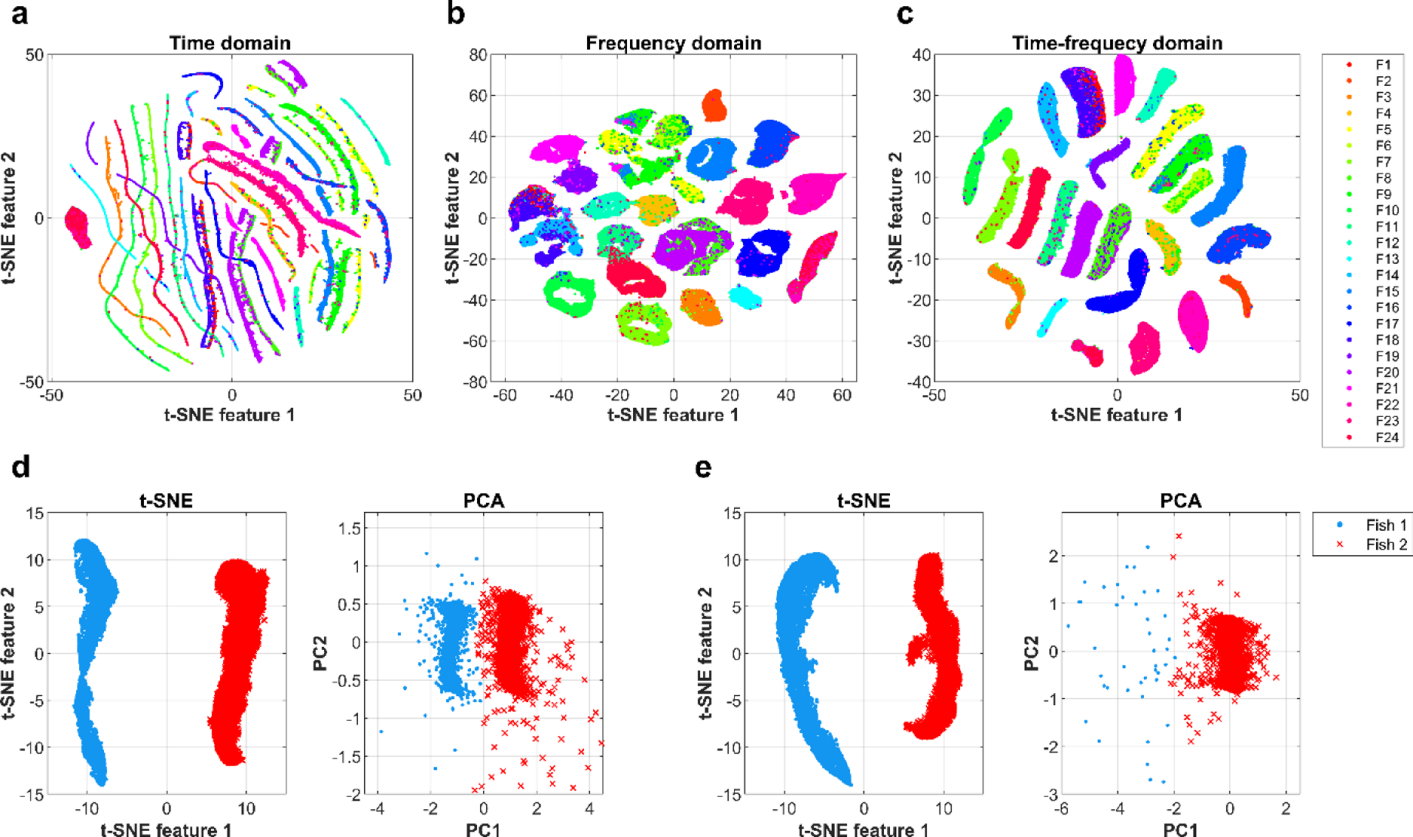
Exploration of inter-individual variability with dimensionality reduction techniques. a-c, Images illustrate outputs from the t-SNE algorithm with EOD features extracted from different domains using Dataset 1. That is: (**a**), original waveforms in the time domain, (**b**), Fourier transform of original waveforms in the frequency domain, and (**c**), coefficients of continuous wavelet transform in the time-frequency domain. A different color label is used for each of the 24 individuals from Dataset (1) (**d-e**), Comparison of the visualizations of time-frequency features utilizing two dimensionality reduction techniques: t-SNE (left) and PCA (right) on recordings from Dataset (2) (**d**), Illustration of a scenario where two distinct clusters can still be observed after applying PCA. (**e**), Illustration of a scenario where the t-SNE algorithm effectively identifies two easily separable clusters, while PCA suggests the data are not easily separable into two classes. The distribution of these two cases was balanced at 50/50.

As shown in Fig. 3a-c, the 2D mappings produced by the t-SNE algorithm demonstrate clearly visible differences in cluster compactness. t-SNE was run with perplexity values ranging from 30 to 120, and the final output was selected based on visual inspection and two clustering quality metrics (average silhouette score – SS, and composed density between and within clusters – CDbw^30^ to ensure the most distinguishable separation of clusters. The time-frequency domain representation, obtained by continuous wavelet transform, resulted in the most distinct and well-separated clusters (SS = 0.50, CDbw = 0.0026), highlighting its effectiveness in capturing inter-individual variability compared to the time (SS = -0.28, CDbw = 0.0014) and frequency domain (SS = 0.37, CDbw = 0.0015) representations. Silhouette plots corresponding to outputs in Fig. 3 are available in the Supplementary material (Figure S1). To determine the optimal perplexity value for the EOD separation applied in subsequent datasets (2–4), EODs from all possible dyadic combinations of 24 individuals were processed using our developed unsupervised approach, which incorporates hierarchical clustering for the classification of t-SNE mapping. Classification performance was assessed using the same set of metrics – accuracy (ACC), Matthews correlation coefficient (MCC), running time normalized by the corresponding number of classified EODs (RT) – that are later employed to compare different separation approaches. Based on these metrics, a perplexity value of 50 was selected for further analyses, see Figure S2 in Supplementary Information.

## Comparison of linear and nonlinear dimensionality reduction methods

In the analysis of the dyadic recordings from Dataset 2, time-frequency features of EODs were extracted and subsequently visualized in a two-dimensional space using t-SNE and PCA. PCA revealed two distinct clusters in 50% of the recordings; however, in the other half, only a single cluster was observed, and hierarchical clustering did not successfully categorize the fish into two groups. In contrast, t-SNE consistently identified distinct clusters in each instance. Figure 3d-e illustrates two types of results generated by t-SNE and PCA alongside the classifications produced by hierarchical clustering.

## Performance of EOD separation approaches

To evaluate the performance of our unsupervised EOD separation approach, we compared it against two supervised approaches: the correlation method and a support vector machine (SVM) classifier. For these purposes, Dataset 2, including the individuals recorded separately and in dyadic combinations, was used. In the correlation-based method, EODs extracted from dyadic recordings were assigned to the individual whose averaged template scalogram from single fish recordings yielded the higher correlation. In the SVM-based method, classifiers were trained on EOD scalograms from individuals and subsequently applied to classify EODs in dyadic recordings involving the same pair of individuals. All results of performance metrics are reported as median values across Dataset 2.

**Fig. 4:**
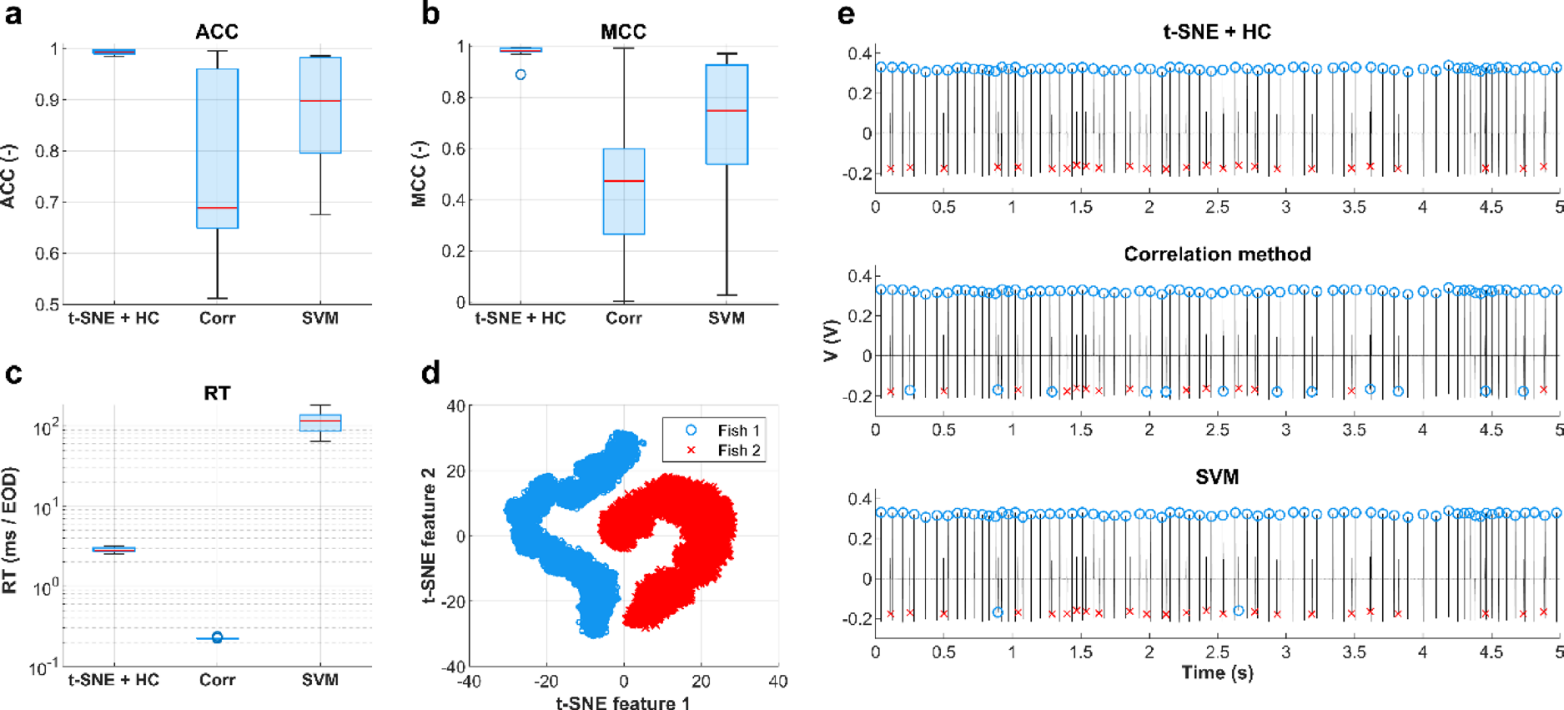
Comparison of EOD separation approaches. (**a**–**c**), Boxplots depicting values of performance metrics (Accuracy (**a**), Matthews correlation coefficient (**b**), and Running time (**c**)) obtained from Dataset 2. A logarithmic scale is used for the running time y-axis to better visualize performance variations. Boxplots illustrate that our unsupervised approach (t-SNE + HC) achieved the highest median ACC and MCC values with small interquartile ranges. (**d**), Result of 2D mapping of feature vectors from a randomly selected recording of dyads obtained using the t-SNE algorithm. Clusters are colored with classification labels from hierarchical clustering corresponding to two individuals. (**e**), Comparison of the results from a classification using (from top) t-SNE + HC approach, correlation method, and SVM classifier on the 5-second segment from the same electric signal as in (**d**). Color markers on EODs correspond to clusters’ color in (**d**). Only the t-SNE + HC approach correctly assigned all EODs on the illustrated signal segment.

Our developed approach was executed with consistent hyperparameter setting, always identifying two distinct and well-separated clusters. It outperformed the correlation method (ACC = 0.688, MCC = 0.472, RT = 0.22 ms/EOD) and the SVM classifier (ACC = 0.898, MCC = 0.747, RT = 115.52 ms/EOD), achieving an accuracy of 0.993 and a Matthews correlation coefficient of 0.981, with classification completed in approximately 2.76 ms/EOD. Figure 4a-c shows the distribution of performance metrics in the form of boxplots. The supervised classifiers achieved high accuracy and Matthews correlation coefficient on some recordings, while performing like a random classifier on others. In comparison, our approach achieved high values of these performance metrics with low interquartile ranges, indicating consistent performance across the recordings. Figure 4d depicts the 2D mapping of feature vectors and their classification using our approach on the randomly selected recording of dyad, with its effectiveness further demonstrated in Fig. 4e. On a selected segment, our approach correctly classified all EODs, while the other two algorithms showed some misclassifications.

Given the large interquartile ranges in the performance of supervised classifiers, we aimed to explore the dependency of their performance on differences in the measured body characteristics of the fish in dyads. No relationship was confirmed, as the correlation analysis did not reveal any significant findings. The summary of results from the correlation analysis is presented in Table 1.

**Table 1:** Correlation of supervised classifiers’ performance with body parameters differences. Values of the pearson correlation coefficient and corresponding *p*-values between the results of performance metrics (Accuracy, Matthews correlation coefficient) of two supervised algorithms and differences in body parameters of fish in a measured dyad. No significant relationship between the algorithms’ performance and differences in physical characteristics was observed.

		Δ Total weight		Δ Total length		Δ Standard length	
		ACC	MCC	ACC	MCC	ACC	MCC
Correlation method	r	0.0868	0.0995	-0.477	-0.415	-0.267	-0.210
	p	0.812	0.784	0.164	0.234	0.456	0.561
SVM	r	0.177	0.186	0.0674	-0.0364	-0.0461	0.162
	p	0.626	0.607	0.853	0.921	0.899	0.654

## Sonification of EOD signal

Understanding how behavior relates to EOD signaling necessitates combining both data modalities into a synchronized representation. To support this process, we introduce two EOD sonification techniques (pulse-wise and FM approach) for initial exploration of this relationship. EODs from the long recording of the dyad (Dataset 3) were classified with our EOD separation approach, and subsequently sonified and integrated into the video recording. Demonstrations of these audio-visual representations are provided as supplementary materials (10.5281/zenodo.14960395). The first example consists of two videos (Video S1 and S2), each containing the sonified signal from one of the two recording channels with preserved information about the amplitude of EODs. These video segments demonstrate how EOD amplitudes fluctuate depending on the fish’s position relative to the recording electrodes, increasing as they approach and decreasing as they move away. The remaining video examples (Video S3 and S4) highlight the potential of sonification techniques in the exploration of fish interactions. In Video S3, the sonification allows us to hear fluctuations in EOD rates while maintaining the precise temporal alignment of individual EODs. On the other hand, video S4 facilitates the examination of variations in EOD rates through evolving melodies, which may give rise to both harmonic and disharmonic structures. We also provide complete audio tracks (S5 and S6), enabling a more comprehensive exploration of EOD interactions themselves over longer temporal scales.

### Effect of ketamine on EOD signaling and behavior

Focusing on *G. petersii* as a potential novel animal model for schizophrenia research, our analyses specifically aimed to elucidate how electrical activity, as a primary modality, influences behavior and how this relationship may be perturbed by ketamine. To achieve this, signals from Dataset 4, comprising dyadic recordings under both Baseline and Ketamine treatment conditions, were initially processed using our EOD separation approach. For each individual, the total number of EODs was extracted, and locomotion, as a behavioral metric, was quantified by the total distance moved based on video tracking (LoliTrack version 4). A linear mixed-effects model was fitted to assess the influence of EOD signaling, treatment condition (Baseline, Ketamine), and experimental group (Active, Control) on locomotion. Random intercepts were included to account for repeated measurements within individuals and dyadic pairing in the experimental setup. The response variable was Box-Cox transformed to meet assumptions of normality and homoscedasticity of residuals.

**Fig. 5:**
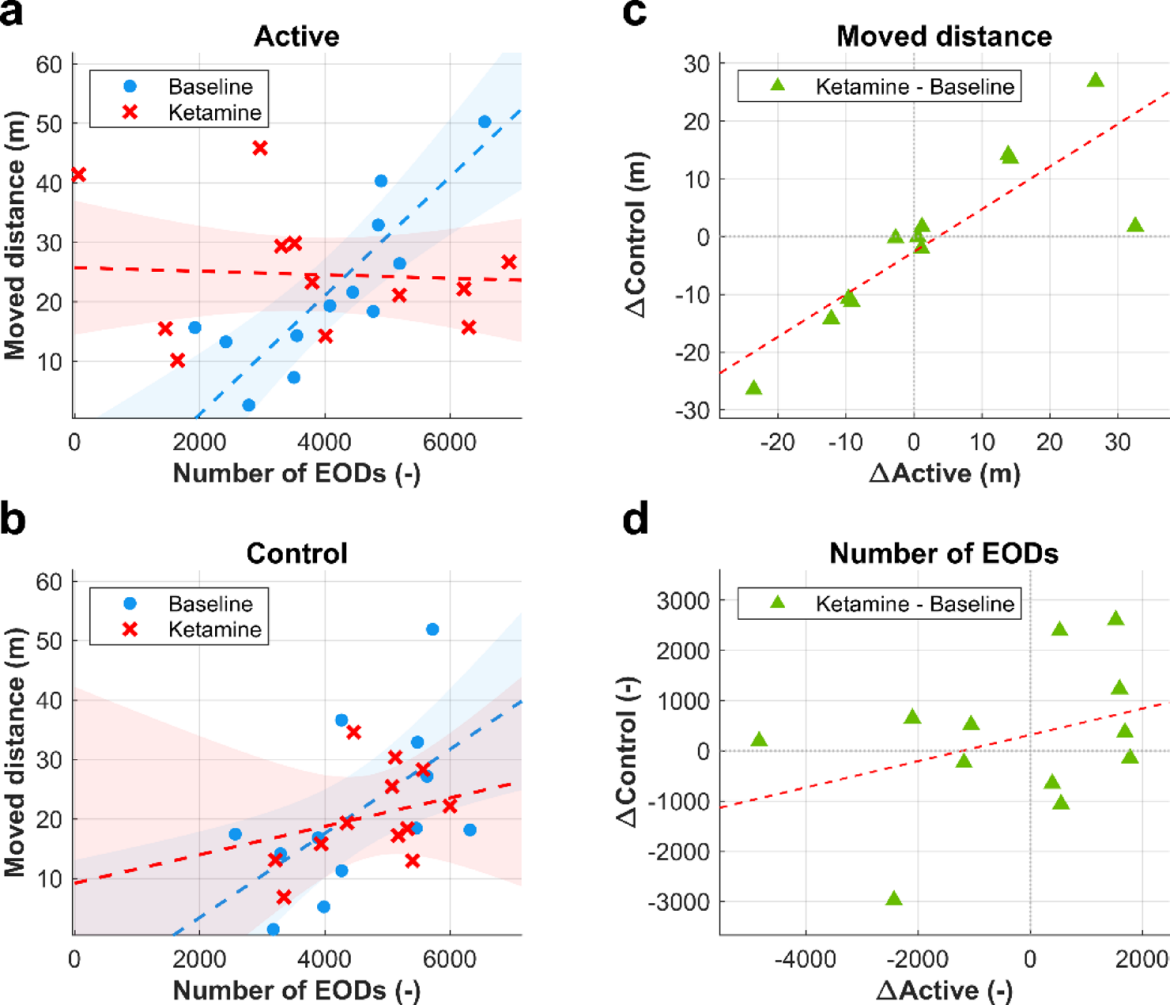
Effect of ketamine on locomotion and EOD signaling. (**a**,** b**), Relationship between the number of EODs and locomotion (total distance moved) for individual fish within the Active (**a**) and Control (**b**) group. Each data point represents the EOD count and distance moved for an individual fish during a single recording session. Data points are shown for both Baseline (blue dots) and Ketamine (red crosses) treatment conditions, with corresponding regression lines and 95% confidence intervals (shaded areas). In the Active group (**a**), a strong positive relationship was observed between the number of EODs and locomotion under the Baseline Condition (*p* < 0.001), which became non-significant after ketamine treatment (*p* = 0.816). In the Control group (**b**), the relationship did not significantly differ between Baseline and Ketamine conditions. **c**,** d**, Correlation analysis of within-subject differences for locomotion (**c**) and the number of EODs (**d**). Each data point represents a difference between the values measured during the Ketamine and Baseline treatment conditions (Δ = Ketamine - Baseline) for a single individual. The x-axis values denote the differences in individuals from the Active group (ΔActive), and the y-axis values denote the differences in their paired conspecifics from the Control group (ΔControl). For locomotion (**c**), a significant positive correlation was observed between ΔActive and ΔControl (*p* = 0.0011), indicating that locomotion differences in ketamine-treated individuals correspond with differences in their paired untreated conspecifics. For the number of EODs (**d**), no significant relationship was confirmed (*p* = 0.499).

A significant three-way interaction between the number of EODs, treatment condition, and experimental group was found (F(1, 15.96) = 5.32, *p* = 0.035), indicating that the effect of treatment condition on the relationship between EOD signaling and locomotion varies across the experimental groups. To further explore the significant three-way interaction, we constructed two separate models for the Active and Control groups. In the Active group, a significant interaction between the number of EODs and treatment condition was observed (F(1, 10.73) = 26.92, *p* < 0.001). Under Baseline condition, locomotion increased significantly with the number of EODs (est. = 9.98e-3, t(14.4) = 5.02, *p* < 0.001), whereas under Ketamine condition, this relationship was markedly weaker and non-significant (est. = -0.29e-3, t(13.5) = -0.24, *p* = 0.816). A direct comparison between treatment conditions confirmed a significant reduction in the effect of the number of EODs on locomotion following ketamine treatment (t(11.4) = -5.02, *p* < 0.001, Fig. 5a). In contrast, within the Control group, the interaction between the number of EODs and treatment condition was not statistically significant (F(1,16.57) = 1.38, *p* = 0.257, Fig. 5b), indicating that the relationship between EOD signaling and locomotion remained stable across treatment conditions. However, a significant main effect of number of EOD on locomotion was observed (F(1,15.42) = 5.52, *p* = 0.032), suggesting a general positive association between EOD signaling and locomotion in this group, irrespective of treatment condition.

To examine the relationship between the ketamine-induced changes in individuals from the Active group and their untreated conspecifics from the Control group, correlation analysis was performed on the within-subject differences between Ketamine and Baseline conditions in both locomotion and EOD signaling. A significant positive correlation was revealed in the analysis of differences in locomotion (*r* = 0.84, *p* = 0.0011), indicating that changes in locomotion observed in ketamine-treated individuals were associated with concurrent changes in their paired untreated conspecifics (Fig. 5c). In contrast, no significant correlation was observed in the analysis of differences in EOD signaling (*r* = 0.36, *p* = 0.499), suggesting that ketamine-induced alterations in treated individuals were not mirrored in their untreated conspecifics (Fig. 5d).

## Discussion

The main result of this study is an effective approach for capturing inter-individual variability in EOD shapes by applying a continuous wavelet transform for feature extraction and t-SNE for dimensionality reduction. It is essential for the possibility of a detailed study of the electrocommunication (or even proto-language) of this unique species. The time-frequency representation using scalograms enabled a more accurate differentiation between the 24 individuals recorded separately, compared to the original waveforms in the time domain or their frequency spectra obtained via fast Fourier transform. This was evident from the highly compact and easily distinguishable clusters observed (Fig. 3a-c). Our findings extend previous research focused on the classification between sympatric species of the weakly electric fish *Gymnotus*^31^ by showing that the superiority of time-frequency representation is also observable within the intraspecies identification. The combination of scalograms and t-SNE proved to be a powerful approach in achieving the high classification performance demonstrated in our study.

The performance metrics results demonstrate (Fig. 4a-c) that our unsupervised separation approach, which incorporates hierarchical clustering for classifying the 2D mapping obtained from t-SNE, outperformed the correlation method and SVM classifier. One of the key advantages of this approach is that it eliminates the necessity of recording individual fish separately to create a training dataset, thereby reducing the overall number of recordings needed for the experiment and offering a novel alternative to existing supervised algorithms for EOD separation^26–29^. This simplification makes the experimental procedure more efficient and, primarily, minimizes stress for the animals involved. Also, we avoided the time-consuming training phase of the SVM or any other advanced supervised algorithm that could be potentially used for classification. Additionally, our approach generates visual representations of high-dimensional data, which can serve as an initial indication of successful separation. For instance, the presence of two distinct clusters in the 2D mapping may suggest that the separation approach correctly distinguished the signals from two fish, providing a useful first step in the validation process.

In this study, hierarchical clustering was employed for classification, primarily because it allowed an explicit definition of the number of desired clusters with minimal need for complex parameter tuning. Given the nature and structure of our datasets, this method was both effective and straightforward. Our current methodology primarily focuses on separating EODs from dyads. This emphasis is justified as it prepares the groundwork for more detailed and fundamental analyses of communication patterns, which are less explored in this specific context. Whether our separation method can be extended to larger datasets involving triads or small groups remains an open question. Such scenarios would involve substantially more data, requiring greater robustness from the analytical pipeline. Consequently, future studies will likely need to incorporate more advanced algorithms, such as HDBSCAN or spectral clustering, which might be better suited to handling increased complexity and identifying complex cluster shapes in multi-individual datasets.

It is important to note that both supervised algorithms exhibited large interquartile ranges in the performance metrics examined (Fig. 4a-c). While the algorithms demonstrated high performance on some recordings, their behavior was similar to random classifiers on others, suggesting that the classification accuracy of supervised algorithms depends on the specific combination of individuals. The observation is further supported by the finding that PCA revealed two clusters in half of the cases and one cluster in the other half (Fig. 3d, e). Given this performance variability, the potential correlations between the differences in body characteristics of the recorded fish and classification outcomes were explored, but no significant relationship was found (Table 1). These results indicate that inconsistent performance is not simply explained by these readily observable measurements, and the reason for the high variability in the separation success/failure remains unclear. Nevertheless, this finding highlights the effectiveness of our unsupervised separation approach, which performed consistently across all recordings.

One of the limitations of our proposed EOD separation approach is the need for manual assignment of separated signals to the corresponding individual (e.g., treated or control). Although it effectively distinguishes two clusters, it does not assign each cluster to a specific fish. This process is relatively straightforward, as the polarity of the EODs is influenced by the fish’s position in the aquarium, allowing accurate identification. However, the processing pipeline still requires user intervention. In further development, incorporating fish tracking data into the classification process^28^ could improve accuracy and eliminate the need for manual assignment, thus enabling a fully automated procedure for signal separation. Another limitation of our approach is its suitability for offline analysis only, as it is not designed for real-time processing.

The primary objective of our work is to contribute to a better understanding of the exceptional electrocommunication abilities of *G. petersii* in studying neural mechanisms underlying communication deficits, particularly in schizophrenia and other psychiatric disorders associated with speech impairments^13,18,32^. To demonstrate it, we performed an experiment on pairs of fish, one of which had schizophrenia modelled using ketamine, and utilized our EOD separation approach. Our results showed that ketamine administration specifically altered the intrinsic relationship between EOD signaling and locomotion, a pattern that was not observed in their untreated paired conspecifics. These findings correspond with our previous single fish recording study documenting that ketamine attenuated the relationship between EOD signaling and locomotion^13^. Crucially, the current study extends these observations to a dyadic setting. Apart from the confirmed alteration of this relationship, the changes in the control fish represent novel findings. Specifically, we confirmed that the locomotion changes in control fish co-varied with the ketamine-induced locomotion changes in active fish, suggesting a social transfer of general behavioral state. Conversely, ketamine-induced EOD alteration of the active fish did not show a correlated response in the EODs of the control fish, which may be interpreted as a substantial deficit of communication in this dyad. However, while the corresponding effect of ketamine on locomotor activity in both fish is evident in our data, we cannot draw such a clear conclusion regarding the effect of ketamine on electrocommunication, as distinguishing electrocommunication from electrolocation based solely on this metric remains challenging. Future research incorporating more detailed characterization of social behavioral patterns will be necessary to clarify the communicative nature of altered EOD activity. At this point, a multimodal analysis will be required to characterize EODs in the contexts of social interaction and navigation.

Our observations have both theoretical and practical implications. They allow us to assess the influence of an individual’s disease model on the behavior of other group members, which is important for the possibility of modeling the role of social factors on disease expression. Practical implications may lie in the use of testing novel treatments. It can be speculated that the optimal antipsychotic should not only normalize the ketamine-altered relationship between locomotion and EODs in intoxicated individuals^13^ but also enable the emergence of a functional inter-individual EOD association between the fish in the dyad, serving as a proxy for treatment-related restoration of effective communication and information transfer.

Together, the ability to accurately separate EOD signals without prior training data represents a significant advancement in electrocommunication research and may serve as an initial step toward studying social interactions both in basic research of weakly electric fish and in preclinical modeling of psychiatric diseases. Sonification techniques can improve this process by providing a more intuitive way to explore the temporal dynamics of EOD sequences and their correlation with fish behavior, facilitating the development of an EOD-behavior state space. With these methodological innovations, future electrocommunication studies can be significantly enhanced, providing deeper insights into social interactions and establishing *G. petersii* as a promising model organism in applied neuroscience and psychotropics development.

## Methods

### Animals

A total of 46 fish of the *G. petersii* species were obtained from a local distributor, VIVARIUM Melnik (Melnik, Czech Republic). The sex of the individuals was not determined since sexual dimorphism in *G. petersii*, manifesting by alteration of EODs, is season-dependent and prolonged captivity can reverse these differences^33,34^. The 126 L (60 × 60 × 35 cm) experimental aquarium was refilled with 30 L of fresh water before each experiment. The water conductivity was 267.21 土 27.67 µS/cm, temperature 24.56 土 0.59 °C, and pH 7.41 土 0.24. All experiments were performed in the dark and in the dark part of the LD regime. The experimental aquarium was illuminated with red light with an intensity of 9 lx on the water surface, due to the high sensitivity of the cone cells of *G. petersii* to deep-red wavelengths, simulating the red-dominated turbid waters characteristic of its natural habitat^35,36^. The study was approved by the ethics committee of Charles University (registration numbers MZE-18134/2019–19014 and MZE-30994/2024–13143) and was in accordance with the local legislation and institutional requirements. The study was reported in accordance with the ARRIVE guidelines.

### Experimental design

Four different datasets were used in this study. Dataset 1 was acquired by recording 24 individuals (total weight = 10.37 土 2.76 g, standard length = 7.85 土 1.24 cm, total length = 12.30 土 1.13 cm) separately to obtain 10-minute recordings. This dataset was used to explore the variability in EOD shapes between individuals and to discriminate the most effective representation of EODs based on these findings. Dataset 2 was used to develop and validate the most appropriate approach for separating signals from two free-swimming individuals. Five individuals (total weight = 7.08 土 1.46 g, standard length = 7.06 土 0.62 cm, total length = 11.37 土 0.73 cm) were first recorded for 15 min separately to obtain training data for the supervised classifiers (first part). Subsequently, all possible combinations of pairs were recorded, resulting in 10 recordings of dyads (second part). Six minutes of each dyad recording were extracted to evaluate the classifiers’ performance. Dataset 3 comprises a single long dyadic recording (45 min), acquired for the EOD classification and demonstration of EOD sonification approaches for exploring patterns in electric activity. Dataset 4 included 15 individuals (total weight = 6.76 土 2.67 g, total length = 10.63 土 1.01 cm) and served for the demonstration of our EOD separation approach in social experiments related to schizophrenia research. This dataset consisted of 24 dyadic pairings between pharmacologically treated individuals (Active group; *n* = 12) and untreated conspecifics (Control group; *n* = 3), which were used as a social factor. To minimize the impact of individual variability among conspecifics, each individual from the Control group was paired with multiple individuals from the Active group. Baseline recordings were obtained with both members of each dyad in an untreated state (12 recordings), followed by subsequent recordings wherein the individuals from the Active group were treated with ketamine. Pharmacological manipulations followed protocols established in our previous study^13^. Briefly, control fish were transferred directly to the experimental aquarium, and active fish were transferred for 15 min into a 2 L dark tank filled with 30 mg/l ketamine water^16^. For each of the 24 recordings, a 5-minute segment was selected for analysis.

### Data acquisition

EODs were recorded using the same experimental setup established in our previous studies^13,32^. A specialized data acquisition system comprising three hardware layers: sensor electrodes, an amplifier, and a data acquisition unit. The first layer consisted of Ag electrodes, originally designed for electroencephalographic recording, with four active bipolar-connected electrodes positioned at each corner of the experimental aquarium and a fifth reference electrode in its center. All electrodes were submerged 2 cm below the water surface. Subsequently, the signal was amplified using an instrumental amplifier with a gain of 10. The amplified output was directed to a National Instruments USB-6003 DAQ unit with a sampling rate of 50,000 samples per second and 16-bit resolution for each channel. Data were displayed and stored on a computer with a National Instruments application. Illustration of the experimental aperture, data acquisition, and subsequent processing of recorded EODs is depicted in Fig. 1. The entire acquisition system was synchronized with the 1.3 MPx infrared recording camera (IDS Imaging Development Systems GmbH, Germany) using a common clock cycle to enable accurate synchronization of EOD signal acquisition with digital image capture from an infrared camera. The video recordings were analyzed by LoliTrack version 4 (Loligo Systems, Denmark) to obtain the fish’s moved distance as a parameter of locomotor activity.

### EOD preprocessing and extraction

Recorded signals were analyzed using MATLAB 2023b. The signal isolines approximated through median filtering were subtracted from the signals to eliminate the baseline drift. Before applying the EOD detection algorithm, the signal was squared to enhance the peak amplitude and ensure that the algorithm accurately identifies the maximum peak of the waveform, since the EOD polarity is influenced by the orientation of the fish relative to the electrodes. EODs were detected using the threshold of 5 mV and a minimum distance of 400 µs between peaks. The threshold for EOD detection was determined based on the estimated noise level from experimental recordings (RMS_noise_ = 0.2 mV). EOD detection was performed on both channels; however, only the EOD with the higher amplitude was considered for the subsequent analysis. EODs were extracted from the signal utilizing 300 µs before and after the absolute maximum peak of the waveform. Since the amplitude of the EOD depends on the size of individuals^37^ and their position in the aquarium, the EODs were normalized by the value of the absolute maximum peak (voltage range of 土 1 V) and eventually stored with reversed polarity to maintain consistency between the orientation of the waveforms.

### EOD representations

According to Crampton et al.^31^, using time-frequency features facilitates the classification of different species of weakly electric fish. For this reason, we applied continuous wavelet transform (CWT) with Morse wavelet (γ = 3, P^2^ = 60, 12 Voices per Octave) to each extracted EOD. The mentioned parameters were selected as they offer a sufficient trade-off between time and frequency resolution. Before the transformation, the EODs were padded with zeros (15 zeros on each side) to achieve higher frequency resolution and eliminate boundary effects. After the transformation, scalograms were normalized by the coefficient with the maximal value and cropped back to a size of 31 samples (~ 620 µs). The resulting coefficients were concatenated into a single feature vector. To confirm the efficiency of time-frequency features, the inter-individual differences in EOD shapes were also examined in the time domain – using original normalized EODs and in the frequency domain – using normalized spectra of EODs obtained with the fast Fourier transform. All the representations utilized for the analysis of inter-individual variability are illustrated in Fig. 2. A total of 235,664 EODs from all 24 individuals in Dataset 1 were extracted and stored in three separate matrices utilizing the described representations (time, frequency, and time-frequency domains).

### Dimensionality reduction

For dimensionality reduction of high-dimensional EOD features from Dataset 1, t-Distributed Stochastic Neighbor Embedding (t-SNE)^38^ was utilized. As a nonlinear method, t-SNE effectively captures the global data structure, given that linear techniques may be insufficient for data lying on nonlinear manifolds. To enhance time efficiency, FFT-accelerated Interpolation-based t-SNE was employed^39^. Quality of clusters was quantified by two metrics – the average silhouette score (SS) as a standard measure of cluster compactness, and CDbw (composed density between and within clusters) index, which is well-suited for evaluating clusters with non-spherical shapes and irregular geometries^30^. Dimensionality reduction with t-SNE also served as an initial step for an unsupervised EOD separation approach.

Results from the t-SNE algorithm obtained from Dataset 2 were compared by visual inspection with the outputs using principal component analysis (PCA), a widely utilized linear dimensionality reduction method. This technique provides an efficient and well-interpretable solution; however, it may not sufficiently uncover the underlying structure of the data.

### Classification

The 2D mappings of recordings from Dataset 2, obtained by the t-SNE algorithm, were classified into two clusters using hierarchical clustering with the average linkage method and Euclidean distance. Hierarchical clustering was selected due to the presence of nonlinearly separable clusters after dimensionality reduction, making linear decision boundary-based algorithms, such as k-means, unsuitable for this task. Our unsupervised approach did not require any prerequisites or training data based on the first part of Dataset 2. To find the optimal perplexity value for the t-SNE algorithm, EODs from all 276 possible dyadic combinations of 24 individuals in Dataset 1 were extracted. These combinations were classified using our described unsupervised approach with different perplexity for t-SNE, and the results were evaluated using metrics that were subsequently applied for performance evaluation of classifiers in Dataset 2 (see Methods: Performance Evaluation). The evaluated perplexity values ranged from 30 to 120, and the optimal perplexity was determined as the one achieving the highest scores according to these metrics, ensuring the best classification performance.

The correlation method and support vector machines (SVM) classifier with radial basis function (RBF) kernel were employed as two supervised approaches for comparison with our developed unsupervised approach. For the correlation method, the average scalograms from the recordings of a single fish in Dataset 2 (the first part) were obtained for each of the two corresponding individuals in the dyadic recording in Dataset 2 (the second part). These averaged scalograms were used as templates for the computation of correlation coefficients with scalograms extracted from dyadic recording. Then, the EOD from dyadic recordings was assigned to the individual with the higher Pearson correlation coefficient value.

For the training phase of the SVM classifier, 5,000 EODs with the highest signal-to-noise ratio, determined by the amplitude of the waveform in the time domain, were extracted from the single fish recordings in the first part of Dataset 2. Subsequently, wavelet coefficient predictors were standardized by the corresponding weighted column mean and standard deviation. For each recorded dyad, a model was trained to recognize the two corresponding fish in the second part of Dataset 2. Hyperparameters of the SVM RBF kernel (γ, C) were optimized using Bayesian optimization with 10-fold cross-validation.

### Performance evaluation

To evaluate the performance of the classifiers, EODs from all recordings of dyads were manually assigned to the corresponding individual based on the amplitude and polarity of the signal relative to the individual’s position within the aquarium. A total of 117,658 EODs were assigned in this manner to create the test dataset. The procedure was performed twice to ensure the reliability of the manual assignment. Although there was a disagreement in approximately 0.5% of the instances, the differences in performance metrics of the algorithms clearly exceeded this level of uncertainty. Therefore, we re-evaluated the ambiguous cases and proceeded with our analysis.

Different EOD separation approaches were evaluated based on the accuracy (ACC), the Matthews correlation coefficient (MCC), and running time normalized by the corresponding number of EODs in each recording (RT). Given the significant variability in the counts of EODs during social interactions, the Matthews correlation coefficient was selected as a performance metric due to its robustness when applied to unbalanced datasets^40^. The duration of the training phase of the SVM algorithm was included in the running time value.

After quantification of the separation performance, Pearson correlation coefficients between the performance metrics and differences in measured body characteristics (total weight, total length, standard length) of recorded individuals in dyads were computed to explore the potential relationship between the classification outcomes and variations in fish’s physical parameters. This analysis was performed on supervised classifiers only, as the performance of the unsupervised approach was consistent across all recordings.

### EOD sonification for application in research

Following the separation of EODs from Dataset 3, two sonification approaches were used to map EODs onto auditory representations. The pulse-wise approach represented individual EODs as brief sinusoidal waveforms with two different frequencies corresponding to two individuals in a dyad. The waveforms were designed to ensure distinct auditory discrimination between EODs from two individuals, despite their short duration. For the frequency modulation (FM) approach, sonification of two continuous harmonic signals was employed. Each fish was assigned a specific carrier frequency, which was then modulated based on the IPIs of the respective fish. This process mapped the temporal evolution of EOD rates into two distinct melodies, allowing for auditory perception of synchrony between the fish’s EOD signals. Detailed methodology for both sonification approaches, including specific parameters, is provided in the Supplementary Information.

### Relationship between EOD signaling and behavior

Firstly, recordings from Dataset 4 were separated using our EOD separation approach. For each individual, the total number of EODs was determined, and locomotion (behavioral metric) was quantified as the total distance moved, extracted from video recordings. To assess how locomotion depends on EOD signaling under specific treatment condition, a linear mixed-effects model was fitted using restricted maximum likelihood with locomotion as the response variable, and number of EODs, treatment condition (Baseline, Ketamine), and experimental group (Active, Control) as fixed effects. The degrees of freedom were estimated using the Satterthwaite approximation method. Random effects were incorporated to account for repeated measurements within individuals and pairing of subjects in the experimental design. To account for potential size-dependent variations in ketamine’s effects, residual body mass, calculated as the residuals from a linear regression of body mass on total length, was included as a covariate.

Significant interactions detected in the linear mixed-effects model were further examined with post hoc analyses to clarify the nature of these effects. Specifically, separate models were fit within each experimental group to estimate the relationship between the number of EODs and locomotion under different treatment conditions. Pairwise comparisons of estimated marginal slopes between Baseline and Ketamine conditions were performed to determine whether the strength or direction of this relationship was altered within each experimental group. Assumptions of residual normality and homoscedasticity for all fitted models were verified through residual diagnostics and appropriate tests. Accordingly, variables were power-transformed where necessary to meet these assumptions. Statistical significance was assessed at an alpha level of 0.05, and *p*-values were adjusted using the Bonferroni-Holm correction method to account for multiple testing. All statistical analyses were performed in R (4.5.0).

Additionally, to explore the relationship between the ketamine-induced changes in behavior and EOD signaling between treated and control fish, correlation analysis was employed. Within-subject differences between measured locomotion in the Ketamine and Baseline conditions were computed for treated individuals and the corresponding control conspecifics. From these associated differences, the Pearson correlation coefficient was calculated to assess the inter-group relationship of locomotion between treated individuals and their control conspecifics. The same analysis was performed in terms of within-subject differences in the number of generated EODs.

## Supplementary Information

Below is the link to the electronic supplementary material.

Supplementary Material 1

Supplementary Material 2

Supplementary Material 3

Supplementary Material 4

Supplementary Material 5

Supplementary Material 6

Supplementary Material 7

## Data Availability

Electric signals from Dataset 2, including manually labeled EODs, are available on Zenodo (https://doi.org/10.5281/zenodo.15667865) to facilitate the evaluation of our developed EOD separation approach. The remaining data used in this study will be made available from the corresponding author upon reasonable request without undue reservation.MATLAB codes for the developed EOD separation approach and EOD sonification are freely available on GitHub (https://github.com/ivanachrtkova/EOD_tools).
