# Supplementary material for: Unsupervised electric signal separation for linking behavior and electrocommunication in Gnathonemus petersii
Source: Sci Rep. 2025 Sep 29;15:33542. doi: 10.1038/s41598-025-18291-5 (PMC12479879; doi:10.1038/s41598-025-18291-5)
Supplement: Supplementary file 1 — Supplementary Material 1 [file 41598_2025_18291_MOESM1_ESM.pdf]

Supplementary Information

Unsupervised Electric Signal Separation for Linking Behavior and Electrocommunication in *Gnathonemus petersii*

Ivana Chrtkova[1,2], Vlastimil Koudelka[1*], Veronika Langova[1,3], Jan Hubeny[1], Petra Horka[4], Karel Vales[1], Roman Cmejla[2], Jiri Horacek[1,3]

1 National Institute of Mental Health, Klecany, Czech Republic
2 Faculty of Electrical Engineering, Czech Technical University in Prague, Prague, Czech Republic
3 Third Faculty of Medicine, Charles University, Prague, Czech Republic
4 Institute for Environmental Studies, Faculty of Science, Charles University, Prague, Czech Republic

***Corresponding author:** Vlastimil Koudelka (vlastimil.koudelka@nudz.cz)

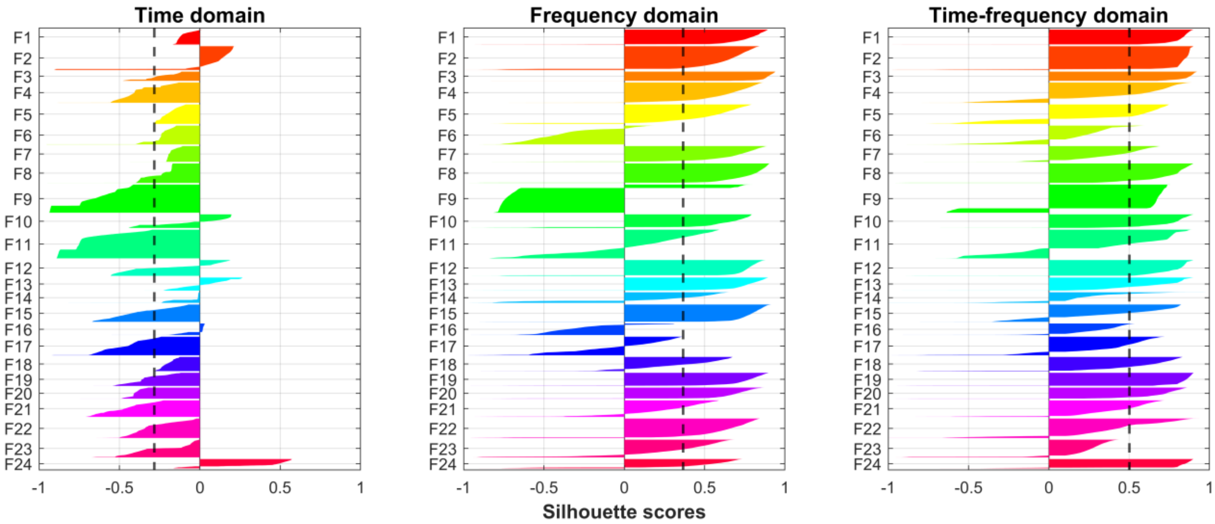

Supplementary figure S1: Silhouette plots for three different domains. Silhouette plots associated with outputs from t-SNE for three different EOD representations in **Figure 3a-c,** with corresponding color coding of individual fish. The black dashed line depicts the average silhouette score: **a**, for the time domain, SS = -0.28; **b**, for the frequency domain, SS = 0.38; **c**, for the time-frequency domain, SS = 0.50. These scores indicate that time-frequency representation captures the highest degree of inter-individual variability.

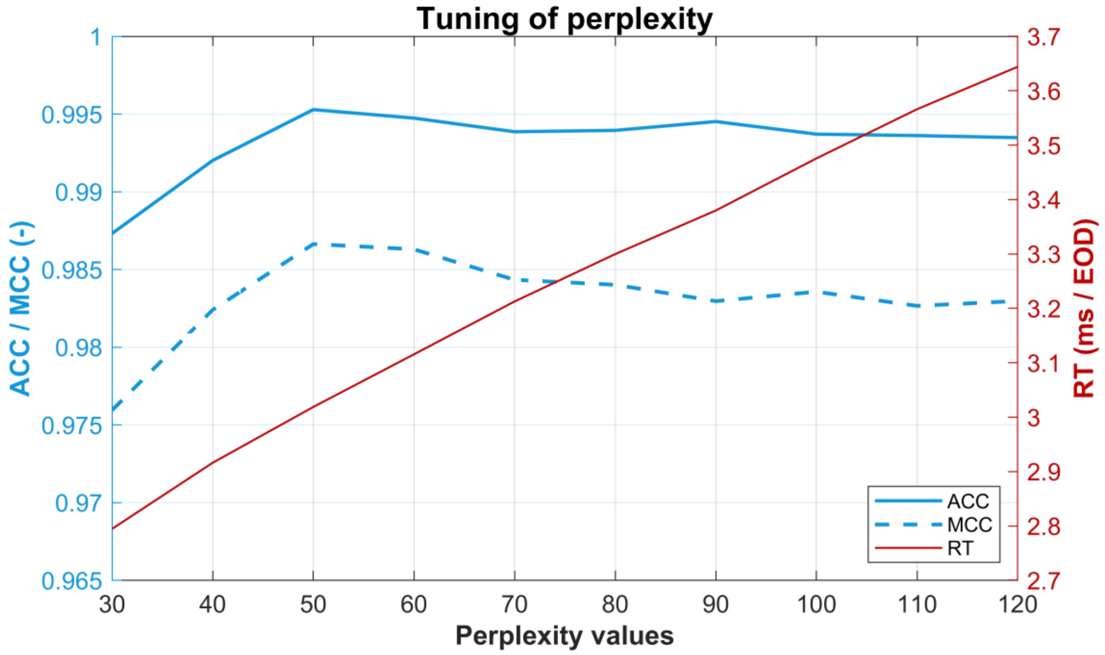

Supplementary figure S2: Tuning of t-SNE perplexity value. Median values of performance metrics (Accuracy, Matthews correlation coefficient, and running time) obtained from our unsupervised EOD separation approach applied to all possible combinations of simulated dyads from Dataset 1. According to these results, the t-SNE perplexity value of 50 was selected for signal separation in subsequent datasets.

Sonification approaches

Due to the short duration of EODs and relatively small inter-individual variability, it would not be possible for the human ear to distinguish two individuals solely based on their sonified EODs. To remediate this point, sinusoidal waveforms with different frequencies for each individual (instead of the original EODs) were inserted at the corresponding instances of the original EODs generated by the fish. The duration of the sinusoidal waveforms was preserved the same as the duration of extracted EODs in previous analyses (620 µs), and amplitudes of the waveforms were weighted by amplitudes of original EODs if maintaining information about the original signal intensity was desired.

Sonified sinusoidal waveforms with such a brief duration are perceived as auditory clicks rather than pure tones, as the pitch of sinusoidal tones is influenced not only by their fundamental frequency, but also by other factors, including duration, intensity, and amplitude envelope [1–4]. Due to this perceptual effect, it was necessary to determine an appropriate combination of frequencies to ensure clear distinguishability between the two sonified waveforms despite their short duration. Experimental testing revealed that a combination of 1.5 and 15 kHz resulted in the most distinct auditory discrimination between the two individuals while preserving the temporal alignment of EODs. Following the separation of EODs from Dataset 3, the signals were sonified as described and integrated into the video recordings.

To explore the structure of EOD signals in two interacting fish, frequency modulation (FM) sound synthesis was applied. First, two distinct and harmonious carrier frequencies, *fc1* and *fc2*, were selected to serve as specific frequency codes for each fish, similar to the approach described earlier: *fc1* was set to 523.3 Hz and 1046.5 Hz (tones C5 and C6), and

fc2 to 392 Hz and 784 Hz (tones G4 and G5). Instead of a pulse-wise approach, continuous sonification of the two harmonic signals was used.

To enhance auditory readability, both harmonic signals were extended by their second harmonics (C5 by C6 and G4 by G5). The carrier frequencies were continuously modulated based on the time interval between two consecutive EODs (IPI) of the fish. Notably, the IPI time series was low-pass filtered using a Butterworth filter with a cutoff frequency of 1 Hz before modulation, ensuring smooth variations in the distinct harmonious frequencies corresponding to different EOD rates. The filtered IPI time series sign was then inverted to provide a direct proportion between the EOD frequency and the carrier frequency. This process mapped the temporal evolution of the two EOD rates, representing the two interacting fish, onto two distinct melodies.

The degree of coupling between the signals, in terms of EOD rates, could be observed as the level of independence between the two melodies. Furthermore, since the two carrier frequencies were chosen to be harmonious, the harmony or disharmony between the two "voices" also played a role. Harmonic states represent situations where the EOD signals of the fish are in perfect synchrony, whereas disharmonic states indicate a lack of synchrony.

References

1. Pollack, I. The Apparent Pitch of Short Tones. *The American Journal of Psychology* **81**, 165–169 (1968).

2. Cohen, A. Further Investigation of the Effects of Intensity upon the Pitch of Pure Tones. *The Journal of the Acoustical Society of America* **33**, 1363–1376 (1961).

3. Hartmann, W. M. The effect of amplitude envelope on the pitch of sine wave tones. *The Journal of the Acoustical Society of America* **63**, 1105–1113 (1978).

4. Rossing, T. D. & Houtsma, A. J. M. Effects of signal envelope on the pitch of short sinusoidal tones. *The Journal of the Acoustical Society of America* **79**, 1926–1933 (1986).
